# Metronomic oral vinorelbine as first-line treatment in elderly patients with advanced non-small cell lung cancer: results of a phase II trial (MOVE trial)

**DOI:** 10.1186/s12885-015-1354-2

**Published:** 2015-05-06

**Authors:** Andrea Camerini, Cheti Puccetti, Sara Donati, Chiara Valsuani, Maria Cristina Petrella, Gianna Tartarelli, Paolo Puccinelli, Domenico Amoroso

**Affiliations:** Medical Oncology, Versilia Hospital and Istituto Toscano Tumori, Lido di Camaiore, Italy

**Keywords:** Elderly, Non-small-cell lung cancer, Metronomic vinorelbine

## Abstract

**Background:**

Metronomic oral vinorelbine could be a safe option for elderly patients with advanced non small cell lung cancer (NSCLC). Metronomic administration of chemotherapy leads to a cytostatic action shifting treatment target from cancer cell to tumor angiogenesis.

**Methods:**

43 chemotherapy naive elderly (≥70 yrs) PS 0-2 patients with stage IIIB-IV NSCLC were prospectively recruited. Median age was 80 yrs (M/F 36/7) with predominantly squamous histology. PS distribution was 0-1(16)/2(27) with a median of 3 serious co-morbid illnesses. Study treatment consisted of oral vinorelbine 50mg three times weekly (Monday-Wednesday-Friday) continuously until disease progression, unacceptable toxicity or patient refusal. Primary endpoints were overall response rate (ORR), clinical benefit (CB – disease response plus disease stabilization >12 weeks) and safety. Health-related QoL (HRQoL) was also assessed with FACT-L V4 scoring questionnaire. We conducted an exploratory time-course analysis of VEGF and thrombospondin-1 (TSP1) serum levels in a subgroup of patients.

**Results:**

Patients received a median of 5 (range 1-21) cycles with a total of 272 cycles delivered. ORR was 18.6% with 7 partial and 1 complete responses; 17/43 experienced stable disease lasting more than 12 weeks leading to an overall CB of 58.1%. Median time to progression was 5 (range 2-21) and median overall survival 9 (range 3-29) months. Treatment was well tolerated with rare serious toxicity. Regardless of severity main toxicities observed were anemia in 44%, fatigue in 32.4%, and diarrhoea 10.5%. FACT-L v4 scores did not significantly vary during treatment. Baseline VEGF levels were lower and showed a rapid increase during treatment in non-responders pts only while TSP1 levels did not change.

**Conclusions:**

Metronomic oral vinorelbine is safe in elderly patients with advanced NSCLC with an interesting activity mainly consisting in long-term disease stabilization coupled with an optimal patient compliance (Eudra-CT 2010-018762-23, AIFA OSS on 26 February 2010).

## Background

Non-small cell lung cancer (NSCLC) is the leading cause of cancer death in Western World [1]. The majority of diagnosis occurs at an advanced stage and available treatments are still unsatisfactory. More than 50% of cases of advanced NSCLC are diagnosed in patients older than age 65 years, and approximately 30% to 40% in patients older than age 70 years [2]. Elderly patients represent an unique setting in which the risk/benefit ratio of treatment should be carefully evaluated. They often present with medical comorbidities and social problems that make the selection of the optimal treatment quite challenging [3]. Chemotherapy with a single agent is an appropriate therapeutic option suitable for a large number of elderly patients with advanced NSCLC [4]. Among available drugs, both infusion and oral vinorelbine (VNR) is widely used with a favorable and foreseeable toxicity profile especially suitable for elderly and/or fragile patients [5,6]. Metronomic chemotherapy (MC) offers the advantage to higher overall drug dose without worsening safety. It contemplates the fractionated, frequent and long term administration of single drug doses without breaks until disease progression or unacceptable toxicity. MC acts as a cytostatic (non-cytotoxic) treatment developed to overcome drug resistance by shifting the therapeutic target from tumor cells to tumor vasculature, thus counteracting tumor regrowth that may occur between chemotherapy cycles [7]. Oral metronomic VNR has been tested in three phase I trials setting 50 mg three times a week (Monday-Wednesday-Friday) as the reference dose. These trials highlighted the excellent safety of this schedule and pointed out its activity [8-10]. Moreover, in the paper by Briasoulis et al. [8] authors found significant treatment-induced variations in some endogenous neo-angiogenesis regulators so steering towards modulation of such pathway to get the anti-cancer effect.

On these grounds, we conducted the MOVE phase II trial to explore the role of oral metronomic VNR as single agent in the first-line treatment of elderly patients with advanced NSCLC.

## Methods

### Eligibility criteria

Chemotherapy naive patients aged 70 years or older able to take oral medications with hystologically or cytologically confirmed, stage IIIB (not suitable for surgery and chemo-radiotherapy) or IV NSCLC according to UICC-TNM 7th edition with RECIST 1.1 measurable disease were eligible for the study. Additional entry criteria included ECOG PS 0–2, a life expectancy of at least 3 months, adequate bone marrow reserve and adequate hepatic and renal function. We excluded patients with previous (within 5 years) or concomitant malignancies, symptomatic brain metastases and activating epidermal growth factor receptor (EGFR) mutations. Concomitant radiotherapy was not allowed. Written informed consent was obtained before study entry and study procedures were in accordance with Helsinki Declaration. This trial received approval by local Ethical Committee (Comitato Etico AUSl 12 di Viareggio) and was registered with Eudra-CT n° 2010-018762-23 and appears on Agenzia Italiana del Farmaco (AIFA) observatory on February 26th 2010. Baseline evaluation included medical history, physical examination, symptom assessment, PS determination, complete blood cell count and serum chemistry. Baseline staging consisted computed tomography (CT) scan of the thorax and upper abdomen. Brain CT and bone scan were reserved to symptomatic patients. Health-related quality of life (HRQoL) was assessed at baseline, during treatment and at study-end by mean of the Italian version of FACT-L v4 questionnaire. We consider the following as serious co-morbid illnesses: Heart disease (previous myocardial infarction, heart failure, valvular heart disease and serious arrhythmias), chronic obstructive pulmonary disease (COPD), diabetes, cerebral or peripheral vascular disease, chronic renal failure, hepatitis and/or cirrosis, hypertension and severe auto-immune diseases.

### Study design and treatment

Oral vinorelbine was administered at the dose of 50 mg (one capsule of 20 mg plus one of 30 mg) three times weekly on Monday, Wednesday and Friday continuously until disease progression, patient refusal or excessive toxicity. Vinorelbine capsules were taken after a meal without chewing or sucking the capsules. No primary prophylaxis with antiemetics was recommended but delivered upon request. In case of diarrhoea loperamide was recommended. Granulocyte colony-stimulating factors were allowed in grade 3 neutropenia with fever lasting ≥3 days or in case of grade 4 neutropenia. The use of erythropoietin was allowed. We consider three weeks as a cycle. Patients took treatment at home. Patients were seen every cycle and complete blood cell count and serum chemistry were performed. Dose adjustment was made as follow: if grade 3/4 hematologic or non-hematologic toxicity or persistent grade 2 toxicity with impact on daily activities occurred at any time during cycle, dose was reduced to 30 mg three times weekly at first occurrence and to 20 mg three times weekly at second occurrence. If grade 3/4 toxicity still occurs treatment was permanently stopped. Patients received any other palliative treatment needed.

### Disease assessment and study objectives

Disease evaluation was performed with chest/upper abdomen CT every nine weeks during treatment. During follow-up disease evaluation was performed every three months. Primary end-points were response rate (RR) (according to RECIST 1.1 criteria), clinical benefit (CB – defined as RR plus stable disease lasting more than 12 weeks) and safety. Secondary end-points were time to progression (TTP), overall survival (OS) and HRQoL. Complete and partial responses were defined according to RECIST 1.1. TTP was calculated from the date of treatment start to the date of first-documented progression or patient death. OS was defined as the time interval between the start of study treatment and death or last follow-up contact. Adverse events were recorded according to the CTCAE v3.0.

### Exploratory VEGF and thrombospondin-1 analysis

Patients who agreed to optional exploratory substudy were required to sign a separate additional informed consent. Peripheral venous blood samples were taken at baseline, every 3 weeks for the first 3 months and then at disease progression. Serum samples were stored at −20°. Vascular endothelial growth factor (VEGF) and thrombospondin-1 (THS1) concentrations were determined using ELISA commercially available kits. Protocols, procedures, and equipment were used according to the manufacturer's specifications. VEGF levels were expressed in pg/ml and TSP1 in ng/ml. Analysis were carried out in duplicate. Exceeded serum was destroied.

### Statistical methods

Given a low-interest response rate (P0) of 10% and a treatment-related response rate of clinical interest (P1) of 25%, an α-error of 0.05 and β-error of 0.2, according to Simon’s Minimax design for two-step phase II trial we aimed to enroll 18 pts at first step. In case of treatment responses >2 the enrolment continued to a total of 43 pts. Study treatment can be considered of clinical interest in case of a total treatment responses >7. Trial accrual started on march 2010 and ended on July 2013. All data were analyzed at a cut-off date of January 2014 representing the disease progression time (and so the end of active treatment) of last study patient. At report time overall survival data are available for all patients. Survival parameters (TTP and OS) were expressed as median and range. In the exploratory VEGF and TSP1 serum level analysis, values were expressed as mean ± standard deviation of and their differences were tested for significance with Student’s *t*-test.

## Results

### Patient characteristics

First-step results were available October 2011. We observed 3 treatment responses with a good safety profile so we kept on enrolment until a total of 43 patients. Baseline study population characteristics are shown in Table 1. Median age was 80 [range 70–92] years. Sex distribution showed a clear predominance of males (M/F 36/7) with squamous cell-histology tumors being the most represented (24/43). ECOG PS 2 patients represented the 62.8% (27 out of 43) of the whole population with a median of 3 [range 0–6] serious co-morbid illnesses. Most frequent co-morbid illnesses were COPD (63%), heart disease (38%) and diabetes (21%).

**Table 1:** **Baseline study population characteristics (n = 43)**

Age (yrs)	
*median (range)*	80 (70 – 92)
Sex (M/F)	36/7
ECOG PS (0/1/2)	0/16/27
Stage (IIIB/IV)	16/27
Smoke (never/past/current)	1/23/19
Serious co-morbid illnesses	
*median (range)*	3 (0 – 6)
Histology (n/%)	
*Squamous cell carcinoma*	24/43 (55.8%)
*Adenocarcinoma*	11/43 (25.6%)
*Large-cell carcinoma*	4/43 (9.3%)
*Undifferentiated*	4/43 (9.3%)

### Drug administration

A total of 272 cycles were given with a median number of cycles of 5 (range 1–21). All patients received at least 1 cycle with 55.8% (24/43) that received at least 5 cycles. One-step dose reduction to 30 mg three times weekly occurred in 7 patients (in all cases after 3 cycles) due to fatigue in 3 patients, nausea in 1 patient and to diarrhoea in 3 patients. Only one patient required two-step dose reduction to 20 mg three times weekly due to grade 3 diarrhoea. After dose reduction the patients did not experience any further significant toxicity. Dose delay of few days occurred in 5 patients for a total of 10 cycles and it was not related to grade 3/4 toxicity but to patient personal preferences. Treatment compliance was high.

### Efficacy

All patients received at least 1 treatment cycle and, at report time, all of them experienced disease progression and were consequently evaluable for both efficacy and safety analysis. Four patients are still alive. We observed 7 partial responses and one complete response in a patient with bilateral lung disease resulting in an overall RR of 18.6%. Moreover, 17/43 (39.5%) showed disease stability lasting more than 12 weeks with a global CB of 58.1% (Table 2). Survival analysis demonstrated a median TTP of 5 (range 2–21) and an OS of 9 (range 3–29) months (Table 2). The percentage of alive patients at one year was 37.2% (16 out of 43) with 4 patients alive at two years. The final RR of 18.6% (8 out of 43 patients) met the default clinical interest threshold. Interestingly, 13 out of 43 (30.2%) patients received a second-line treatment and 4 out of 43 (9.3%) a third-line one.

**Table 2:** **Clinical efficacy data at final analysis on 43 patients**

Number of cycles (median - range)	5 [1 - 21]
Treatment response (n - %)	
CR	1/43 – 2.3%
PR	7/43 - 16.3%
SD	17/43 - 39.5%
PD	18/43 - 41.9%
Clinical benefit	25/43 - 58.1%
ORR	8/43 - 18.6%
TTP (median - range)	5 [2 - 21] months
OS (median - range)	9 [3 - 29] months
Percentage of alive patients (n - %)	
year 1	16/43 - 37.2%
year 2	4/43 - 9.3%

CR = complete response; PR = partial response; SD = stable disease; PD = disease progression; ORR = overall response rate; TTP = time to progression; OS = overall survival.

### Toxicity and quality of life

Study treatment was extremely safe. Grade (G) 3/4 toxicities were rare (two episodes of G3 diarrhoea, one of not-febrile G3 neutropenia, one G3 mucositis, one G3 anemia and two G3 fatigue on a total of 272 cycles delivered). Regardless of severity main toxicities observed were anemia in 44%, fatigue in 32.4%, diarrhoea 10.5%, nausea 8%, vomiting 5% (Table 3). There was no treatment-related death and none of the study patients required hospitalization for treatment-related adverse events. Moreover, during treatment no patient required blood or platelet transfusions or intravenous antibiotics and granulocyte colony-stimulating factors were not used. Only the patient that experienced grade 3 not-febrile neutropenia received oral antibiotics prophylaxis for 5 days. FACT-L v4 scores did not significantly vary during treatment.

**Table 3:** **All grade (left column) and grade 3/4 (right column) treatment-related toxicities at final analysis (n = 43)**

Toxicity	All grade	Grade 3-4
*non-hematological*		
Fatigue	32.4%	0.1%*
Nausea	8.0%	0%
Vomiting	5.0%	0%
Diarrhea	10.5%	0.1%*
Mucositis	4.5%	0.1%*
Sensorial neuropathy	2.4%	0%
*hematological*		
Anemia	44.0%	0.1%*
Leukopenia	3.2%	0%
Neutropenia	4.0%	0.1%*

*Rounded to 0.1%.

### VEGF and thrombospondin-1 analysis

**Figure 1:**
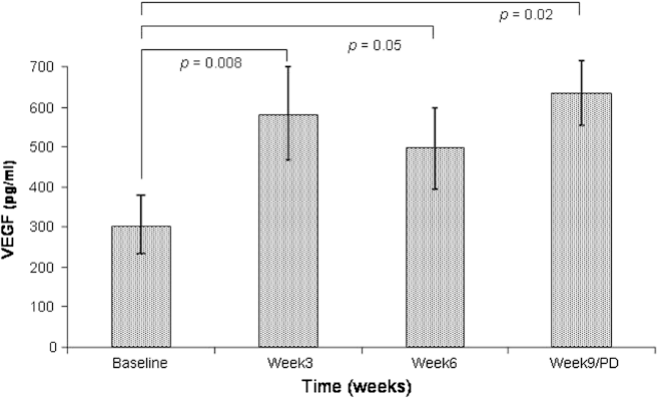
Time course variation in VEGF serum levels in non-responders patients (n = 12).

Serum levels of VEGF and TSP1 were assessed in 28 patients. Baseline VEGF levels significantly differ between non-responders (n = 12) vs responders (including SD >12 weeks) patients (n = 16) (303.8 ± 128.6 vs 660.9 ± 280.4 pg/ml; *p* = 0.04). Time course analysis did not show any significant change in VEGF levels in whole population or in responders patients while in non-responders group (n = 12) we observed a clear increase during treatment until early disease progression (303.8 ± 128.6 vs 579.3 ± 181.2 vs 498.0 ± 211.6 vs 633.4 ± 151.8 pg/ml; *p* = 0.02) (Figure 1). No difference in baseline levels between patients groups or in time course variation were observed for TSP1 levels.

## Discussion

The selection of optimal systemic treatment for elderly patients with advanced NSCLC should rely on both personal (including PS, comorbidities, polypharmacy) and surrounding (familial and social features ) issues making treatment choice as an hard challenge [3]. The best treatment for elderly patients or those with low PS is still debated with single agent chemotherapy being one of the preferred options to treat these patients [11].

Oral vinorelbine could be an attractive option. In fact, with the assumption of an equal efficacy, patients expressed a preference for oral over intravenous chemotherapy [12,13] and the oral formulation could potentially lesser nearly half of the major patient concerns about chemotherapy [14,15].

Our results showed that single agent metronomic oral vinorelbine is a feasible option as first-line treatment in elderly patients with advanced NSCLC. Safety issues are of primary importance in this setting We observed rare grade 3 toxicity making our schedule extremely well tolerated (Table 3). On a total of 272 cycles administered we did not observe any grade 4 toxicity; we only observed two episodes of G3 diarrhoea, one of not-febrile G3 neutropenia, one G3 mucositis, one G3 anemia and two G3 fatigue with and acceptable rate of low grade both hematological and not-hematological toxicities never interfering with treatment, patient dietary intake, daily life or non-study drug administration and without any treatment-related death or hospitalization. Patients did not report any worsening of their QoL scores.

Coupled with the excellent toxicity profile we observed an interesting activity of oral metronomic vinorelbine with an overall RR of 18.6% with 7 partial and one complete responses and a global CB of 58.1%. Survival data were also encouraging with a median TTP of 5 and a median OS of 9 months. Notably, study population is made of "real" elderly patients with a median age of 80 years, a significant number of serious comorbidities and a low PS in more than half of cases.

MILES trial [16] showed that first-line single agent vinorelbine or gemcitabine resulted in an OS ranging from 28 to 36 weeks with a TTP of 17–18 weeks. Previous ELVIS trial [5] demonstrated an absolute survival advantage of vinorelbine plus best supportive care (BSC) over BSC alone with an OS of 28 weeks. In both trials mean age was 74 years with a percentage of PS 2 patients less than 25%. Characteristics of our population are quite different with a median age of 80 years and a proportion of PS 2 patients more than 50%. Oral vinorelbine has been also tested with weekly schedules in 56 chemo-naive NSCLC elderly patients. Grade 3/4 neutropenia was reported in 11/17 out of 56 patients (20/30% of total population respectively) with only 1 febrile neutropenia. Six partial responses and 25 SD were recorded with a median overall survival of 8.2 months [17]. For discussion purposes only, it could be of interest to compare our results with those of the aforementioned ELVIS, MILES (single agent vinorelbine arm) and Gridelli et al. [17] trials in terms of final outcome (approximating OS in weeks). Bearing in mind the different populations, OS was similar (36, 28, 36, 33 and weeks respectively) thus confirming activity of metronomic schedule in real world elderly patients.

In last few years new data on the role of doublet platinum based chemotherapy in elderly advanced NSCLC has emerged. Quoix et al. [18] reported a survival advantage of the carboplatin and paclitaxel doublet versus monotherapy (gemcitabine or vinorelbine). Study population is still different from our with a percentage of PS 2 patient of 27% and a median age of 77; no mention about number of serious comorbid illnesses was reported. Notably, doublet arm was affected by a three-fold increase in toxic deaths and a similar increase in febrile neutropenia and decrease in neutrophil count. Our results cannot be directly compared with French experience. Target population of our study is different from Quoix study. Basically, all study patients were considered eligible to receive platinum as entry criteria while ours did not due to older age, serious comorbidities and low PS. So, as a possible statement, if a patient is deemed fit to platinum doublet he should receive it but, if not, single agent metronomic oral vinorelbine can be an active option. Oral weekly vinorelbine has been widely used in the treatment of NSCLC [19] with a good safety profile. Our data seem to indicate that its metronomic administration can lead to a gain in activity without worsening safety profile. Notably, with the proposed schedule we higher the cumulative dose and, given the dose-effect relationship, we can so suppose to obtain a gain in efficacy. In contrast, delivering such an increased cumulative dose did not affect safety disproving the dose-toxicity relationship. Metronomic administration could so allow us to give an active treatment even in frail patients but still judged suitable for a treatment.

Subgroup analysis of VEGF serum levels gave us some interesting hints. Not-responders patients showed a low baseline VEGF levels respect to responder ones in contrast with Briasuolis et al. [8]. Interestingly, in not-responder patients we observed a rapid increased in VEGF levels kept until disease progression while in responder-ones VEGF levels resulted unchanged. Given the cytostatic/non-cytotoxic action of metronomic treatment interfering with cancer neo-angiogenesis processes [7] we can suppose that in responder patients study treatment can effectively stop tumor growth by limiting neo-angiogenesis and so we do not observe any VEGF level increase. In not-responder ones treatment is uneffective, newly formed endothelial tumor cells spread and we observe an increased VEGF levels contributing to a rapid disease progression.

## Conclusions

This is the first trial testing metronomic schedule in a selected population of elderly advanced NSCLC patients. Our results highlighted the safety of metronomic oral vinorelbine in real world setting of elderly patients with an interesting activity and favourable QOL data. Oral metronomic vinorelbine could so represent a treatment option in elderly patient unfit for a platinum doublet but still suitable for an active treatment.

## Abbreviations

NSCLC: Non-small cell lung cancer
VNR: Vinorelbine
MC: Metronomic chemotherapy
EGFR: Epidermal grow factor receptor
CT: Computed tomography
HRQoL: Health-related quality of life
COPD: Chronic obstructive pulmonary disease
RR: Response rate
CB: Clinical benefit
TTP: Time to progression
OS: Overall survival
VEGF: Vascular endothelial growth factor
THS1: Thrombospondin-1
G: Grade
